# Dissecting Cell Death Pathways in Influenza A Virus Infection: Comparative Insights from Human Models

**DOI:** 10.3390/v18020246

**Published:** 2026-02-14

**Authors:** Ngoc Mai Khoi Nguyen, Alison C. West, Rebecca L. Ambrose, Michelle D. Tate

**Affiliations:** 1 Centre for Innate Immunity and Infectious Diseases, Hudson Institute of Medical Research, Clayton, VIC 3168, Australia; nngu0096@student.monash.edu (N.M.K.N.); alison.west@hudson.org.au (A.C.W.); rebecca.ambrose@hudson.org.au (R.L.A.); 2 Department of Microbiology, Biomedicine Discovery Institute, Monash University, Clayton, VIC 3800, Australia

**Keywords:** influenza A virus, programmed cell death, experimental models

## Abstract

Influenza A virus remains a major global health threat, causing annual epidemics and occasional pandemics. Programmed cell death, including apoptosis, pyroptosis, and necroptosis, with emerging evidence for ferroptosis, plays a dual role in influenza pathogenesis, both limiting viral replication and contributing to immunopathology. Most mechanistic insights have been derived from murine genetic models, which have been invaluable for establishing causal roles of these pathways. However, murine models and cancer-derived cell lines differ significantly from human physiology. This review systematically compares influenza-induced programmed cell death across human-relevant platforms, including primary cells, immortalized non-cancerous lines, co-cultures, organoids, and precision-cut lung slices. The increasing complexity of these models reveals distinct aspects of pathway activation, bystander effects, cell-type vulnerability, and spatial dynamics. We highlight critical divergences between model systems, identify gaps in comparative analyses across viral strains and experimental platforms, and outline future directions leveraging advanced model systems, multi-omics, and functional genomics to enhance translational relevance and guide the development of host-directed therapies.

## 1. Introduction

Influenza A virus (IAV) causes seasonal epidemics and occasional pandemics, resulting in substantial global morbidity and mortality. Seasonal influenza infects an estimated 3–5 million people annually and causes up to 650,000 deaths from severe respiratory complications (WHO) [1]. Despite the availability of vaccines and antiviral drugs, influenza remains a major health burden, particularly during seasons with vaccine mismatch or the emergence of antigenically distinct strains.

Past pandemics illustrate the virus’s capacity for rapid evolution and cross-species transmission. The 1918 “Spanish flu” pandemic caused an estimated 50 million deaths, while later pandemics in 1957 (H2N2), 1968 (H3N2), and 2009 (H1N1) demonstrated how genetic reassortment can generate novel strains capable of efficient human-to-human transmission [2,3,4]. More recently, highly pathogenic avian influenza (HPAI; H5N1 and H7N9) has caused sporadic but severe human infections, raising concerns about future pandemic potential [5,6]. The detection of H5N1 in dairy cattle in 2024 further underscores the unpredictability of influenza ecology and the need for sustained surveillance [7]. Together, these events highlight the persistent pandemic threat posed by IAV and the limitations of control strategies that rely exclusively on viral antigenic stability.

While vaccines and antivirals remain cornerstones for influenza prevention and treatment, their effectiveness is constrained by antigenic drift, resistance, and narrow therapeutic windows [8,9,10]. Moreover, these interventions primarily target viral replication and do not directly address host-driven immunopathology, which is a major contributor to severe disease. This gap is particularly consequential during pandemics, when strain-specific pathogenicity and dysregulated immune responses amplify tissue injury and mortality.

A central determinant of influenza pathogenesis is the induction of programmed cell death (PCD) in infected and bystander cells within the respiratory tract (reviewed in [11,12]). Apoptosis, pyroptosis, and necroptosis are increasingly recognized as central regulators of disease severity, influencing viral replication, inflammatory signaling, and epithelial integrity. Apoptosis generally restricts viral spread with minimal inflammation, whereas lytic forms of PCD, pyroptosis and necroptosis, amplify inflammation and exacerbate lung injury [13]. More recently, ferroptosis has emerged as an additional contributor to influenza-driven inflammation and tissue damage [12,14]. Together, these observations position PCD as a critical interface between antiviral defenses and immunopathology, and a promising but underexplored target for host-directed therapeutic intervention.

Integrating murine in vivo models and human ex vivo and in vitro systems is essential for advancing such therapeutic strategies. Despite considerable progress, significant gaps remain. Many mechanistic studies rely on cancer-derived epithelial cell lines with altered immune signaling or on mouse-adapted viral strains that diverge from natural human infection dynamics [12]. Although these models are experimentally tractable and informative, they incompletely capture the complexity of human respiratory physiology. Comparative analyses across human-relevant systems, including primary epithelial cells, immortalized non-cancerous lines, and advanced models such as organoids and precision-cut lung slices, remain limited. Even fewer studies systematically assess influenza strain-specific differences in PCD responses, despite strong evidence that viral genotype profoundly influences host cell fate decisions.

Although influenza B virus also contributes substantially to the annual disease burden, mechanistic studies of PCD during influenza B infection are comparatively scarce. Accordingly, this review focuses primarily on IAV, which poses the greatest clinical and pandemic threat due to its antigenic diversity and propensity for reassortment. We synthesize current understanding of how IAV engages distinct PCD pathways, their molecular crosstalk, and how pathway balance shapes disease severity. Critically, we compare findings across human-relevant experimental platforms and murine models to identify species-specific divergences and inform translational therapeutic strategies.

## 2. Programmed Cell Death During IAV Infection

**Figure 1:**
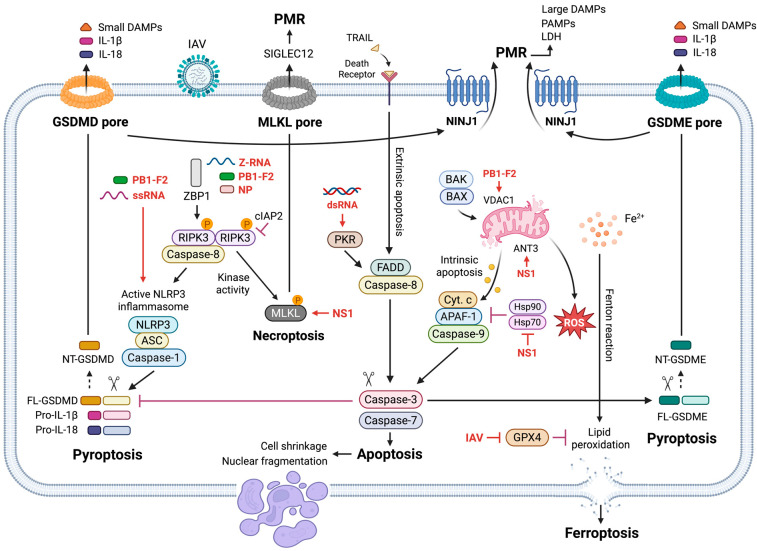
Interplay among programmed cell death pathways triggered by IAV infection. IAV engages intrinsic and extrinsic apoptosis, necroptosis, pyroptosis, and ferroptosis through a network of viral-host interactions. Intrinsic apoptosis is initiated by cellular stressors, including IAV proteins NS1 and PB1-F2 interacting with mitochondrial voltage-dependent anion channel 1 (VDAC1) and adenine nucleotide translocase 3 (ANT3), respectively. NS1 also inhibits anti-apoptotic chaperones (heat shock protein (HSP)70/90), promoting mitochondrial dysfunction. Activation of Bax and Bak permeabilizes the outer mitochondrial membrane, enabling cytochrome *c* (Cyt. c) release and apoptosome formation with APAF-1, which activates caspase-9 and subsequently caspase-3/7 to execute apoptosis. ZBP1 senses viral Z-RNA and interacts with viral proteins PB1-F2 and NP, recruiting RIPK3 to form a multifaceted signaling platform that can induce extrinsic apoptosis, necroptosis, and pyroptosis in parallel. While cellular inhibitor of apoptosis protein 1/2 (cIAP1/2) promotes cell survival, IAV infection shifts this balance toward necroptosis, driven by RIPK3-mediated phosphorylation and oligomerization of MLKL. NS1 enhances MLKL oligomerization, and in human cells, SIGLEC12 mediates terminal PMR downstream of MLKL. Extrinsic apoptosis is initiated by activation of death receptors (e.g., death receptor 4/5 (DR4/5)) by ligands such as TRAIL, leading to formation of the FADD-caspase-8 complex. Caspase-8 undergoes autocleavage and activates caspase-3/7, committing cells to apoptosis and providing crosstalk with intrinsic pathways via Bid cleavage. Pyroptosis is executed through gasdermin-mediated pore formation via two principal routes. IAV components, including ssRNA and PB1-F2, activate the NLRP3 inflammasome, resulting in caspase-1 activation and maturation of IL-1β and IL-18. Caspase-1 also cleaves full-length gasdermin D (FL-GSDMD) to generate the pore-forming N-terminal fragment (NT-GSDMD). Alternatively, during apoptosis, caspase-3 cleaves full-length gasdermin E (FL-GSDME) into its active N-terminal fragment (NT-GSDME) and simultaneously cleaves GSDMD into an inactive p43 fragment. Active GSDMD and GSDME oligomerize to form membrane pores that permit release of small damage-associated molecular patterns (DAMPs) and cytokines. Accumulation of gasdermin pores ultimately triggers NINJ1-mediated PMR, enabling release of large DAMPs, PAMPs, and lactate dehydrogenase (LDH). Ferroptosis, driven by iron-dependent lipid peroxidation and impaired GPX4, acts independently of caspases, gasdermins, and MLKL. Together, these integrated pathways shape the magnitude and inflammatory nature of cell death during IAV infection.

IAV engages multiple PCD pathways in respiratory tissues, and the timing, magnitude, and balance among these pathways strongly influences viral fitness, inflammation, and tissue injury (reviewed in [12]). Rather than acting in isolation, these pathways operate within a dynamically regulated network that governs the transition from early viral containment to inflammatory epithelial damage. As illustrated in Figure 1, IAV can initiate apoptosis through intrinsic (mitochondrial) and extrinsic (death receptor) routes; activate pyroptosis via inflammasome-dependent gasdermin D (GSDMD) or caspase-3-dependent gasdermin E (GSDME); trigger necroptosis through a Z-DNA binding protein 1 (ZBP1)–receptor-interacting protein kinase 3 (RIPK3)–mixed lineage kinase domain-like pseudokinase (MLKL) signaling axis; and promote ferroptosis via iron-dependent lipid peroxidation.

Mechanistic insights into these pathways derive from a combination of human cell culture systems and genetically modified murine models. Across these systems, apoptosis is non-lytic and typically protective, whereas pyroptosis, necroptosis, and ferroptosis are lytic, pro-inflammatory, and frequently associated with lung injury [12,13]. We next describe the core signaling mechanisms underlying each PCD pathway during IAV infection, followed by an integrated discussion of their crosstalk and context-dependent roles in viral control versus immunopathology.

### 2.1. Apoptosis: Intrinsic and Extrinsic Pathways

Apoptosis is the most extensively characterized form of PCD during IAV infection and it is typically immunologically silent. It is characterized by cell shrinkage, chromatin condensation, DNA fragmentation, poly(ADP-ribose) polymerase (PARP) cleavage, and the formation of apoptotic bodies that are efficiently cleared by phagocytes [15]. Across in vitro and in vivo systems, including genetic knockouts, overexpression systems, and pharmacological inhibition, disruption of apoptotic signaling often enhances viral replication and worsens disease outcomes [16,17,18], supporting apoptosis as an important antiviral defense mechanism. However, the functional impact of apoptosis during IAV infection is highly dependent on pathway engagement, timing, and cellular context and certain apoptotic programs can contribute to immunopathology under specific conditions.

#### 2.1.1. Intrinsic (Mitochondrial) Pathway

The intrinsic apoptotic pathway is activated by intracellular stress signals that arise during IAV infection, including viral protein accumulation, oxidative stress, and DNA damage (Figure 1) [19]. This pathway is regulated by the Bcl-2 family proteins, which include anti-apoptotic members (Bcl-2, Mcl-1, and Bcl-XL), BH3-only initiators (Bid and Bim), and pro-apoptotic executioners (BCL2-associated X (Bax) and BCL2 antagonist/killer (Bak)) [20]. Upon activation, Bax and Bak oligomerize at the mitochondrial outer membrane, inducing mitochondrial outer membrane permeabilization (MOMP) and releasing cytochrome *c*. Cytochrome *c* associated with apoptotic protease-activating factor 1 (APAF-1) to form the apoptosome, leading to caspase-9 activation and subsequent cleavage of executioner caspases-3, -6, and -7 [21].

IAV modulates intrinsic apoptosis in a temporally controlled manner. Early during infection, NS1 suppresses pro-apoptotic signaling to prolong host cell survival and maximize viral replication [22]. At later stages, apoptosis is facilitated as viral replication completes, with viral components, including PB1-F2, NS1, NP, and M1, contributing to mitochondrial dysfunction [22,23,24,25,26,27]. In particular, PB1-F2 directly interacts with mitochondrial proteins such as voltage-dependent anion channel 1 (VDAC1) and adenine nucleotide translocase 3 (ANT3), while other viral proteins antagonize host anti-apoptotic mechanisms, including Hsp70 and Hsp90-mediated cryoprotection, thereby permitting caspase activation and apoptotic cell death. This temporal switch enables efficient viral replication while facilitating controlled cell death and viral dissemination.

#### 2.1.2. Extrinsic (Death Receptor) Pathway

Extrinsic apoptosis is initiated by engagement of death receptors, including Fas, tumor necrosis factor (TNF) receptor 1 (TNFR1), and death receptors 4 and 5 (DR4/DR5), by their respective ligands such as Fas ligand (FasL), TNF, and TRAIL (Figure 1) [28,29]. Ligand binding induces receptor trimerization and recruitment of adaptor proteins, including FADD and TRADD, leading to the assembly of death-inducing signaling complexes (DISCs) and activation of initiator caspase-8. Active caspase-8 directly cleaves executioner caspases and amplifies intrinsic apoptotic signaling through Bid cleavage [30,31], thereby linking extrinsic and intrinsic apoptotic pathways.

TNFR1 signaling exemplifies the regulatory complexity of extrinsic apoptosis through the formation of spatially and compositionally distinct signaling complexes. Following TNF binding, membrane-associated Complex I, comprising TNFR1, TRADD, RIPK1, TRAF2, and the E3 ubiquitin ligases cIAP1/2 and LUBAC, promotes NF-κB and MAPK activation, driving pro-survival and inflammatory gene expression. In this context, K63- and Met1 (linear)-linked ubiquitination of RIPK1 stabilizes Complex I and prevents cell death signaling [32].

When RIPK1 ubiquitination is disrupted, through deubiquitination, loss of cIAP1/2 or LUBAC activity, or cellular stress, RIPK1 dissociates from the receptor and assembles cytosolic death-inducing complexes. Formation of Complex IIa, consisting of RIPK1, TRADD, FADD, and caspase-8, leads to caspase-8 activation and execution of extrinsic apoptosis [31,33]. In parallel, under conditions where caspase-8 activity is inhibited or insufficient, RIPK1 can associate with RIPK3 and MLKL to form Complex IIb (the ripoptosome), diverting signaling toward necroptosis [29,34,35].

During IAV infection, infected epithelial and immune cells produce elevated levels of TNF and TRAIL, which can activate death receptor signaling in both infected and neighboring bystander cells [36,37]. In contrast to intrinsic apoptosis, excessive activation of death receptor-mediated pathways can exacerbate immunopathology. Blockage of TRAIL using neutralizing antibodies [38] or inhibition of Fas signaling with decoy receptors [39] improves survival in mice infected with mouse-adapted PR8 H1N1, highlighting a pathological role for dysregulated extrinsic apoptotic signaling during severe influenza.

### 2.2. Pyroptosis: Gasdermins D and E and NINJ1

While apoptosis generally limits inflammation, IAV infection also engages pyroptosis, a lytic and highly inflammatory form of PCD characterized by plasma membrane rupture (PMR) and release of intracellular contents, including damage-associated molecular patterns (DAMPs), pathogen-associated patterns (PAMPs), and inflammatory cytokines (Figure 1) [40,41]. Hallmarks of pyroptosis include secretion of IL-1β and IL-18 and release of cytosolic proteins such as lactate dehydrogenase (LDH) and HMGB1. Pyroptosis is executed by gasdermin family members, primarily GSDMD and GSDME [42]. Genetic ablation of either GSDMD or GSDME improves survival, reduces lung pathology, and lowers viral burden during IAV infection [43,44,45,46], implicating pyroptosis in both immunopathology and viral dissemination.

#### 2.2.1. GSDMD-Mediated Pyroptosis

GSDMD is the most extensively studied pyroptotic effector [41]. It is activated downstream of inflammasomes. Upon activation of inflammasomes such as NLRP3, the adaptor ASC recruits and activates caspase-1, which cleaves GSDMD at Asp275 (human), releasing its pore-forming N-terminal fragment (Figure 1) [47]. In addition, noncanonical inflammasome signaling via caspase-4 and caspase-5 can also cleave GSDMD [48]. Oligomerized GSDMD inserts into the plasma membrane to form pores that permit cytokine release and ion influx, ultimately leading to PMR. Cleaved caspase-1 and GSDMD have been detected in IAV-infected epithelial cells [43,44,49], as discussed in more detail in later sections.

#### 2.2.2. GSDME-Mediated Pyroptosis

GSDME mediates an alternative pyroptotic pathway downstream of apoptosis. Active caspase-3 cleaves GSDME at Asp270, generating an N-terminal fragment that forms membrane pores similar to those formed by GSDMD (Figure 1) [50]. In cells that express GSDME, apoptotic stimuli can therefore be converted into a lytic, inflammatory form of cell death. Caspase-3-dependent GSDME activation has been observed in IAV-infected bronchial epithelial cells [43,51].

#### 2.2.3. Terminal Membrane Rupture: NINJ1

Ninjurin-1 (NINJ1) mediates the terminal step of pyroptosis by driving catastrophic PMR following gasdermin pore formation (Figure 1) [52]. NINJ1 oligomerization enables the release of large DAMPs, PAMPs, and LDH, thereby amplifying inflammatory signaling. NINJ1-dependent PMR has been implicated in lung injury during murine IAV infection [53].

### 2.3. Necroptosis: RIPK1/RIPK3/MLKL Signaling

In parallel with pyroptosis, IAV activates necroptosis, a caspase-independent, lytic PCD pathway mediated by the RIPK1-RIPK3-MLKL axis (Figure 1). When caspase-8 activity is inhibited or insufficient, RIPK3 phosphorylates MLKL, triggering MLKL oligomerization and translocation to the plasma membrane, where it disrupts membrane integrity and causes cell rupture [54]. This lytic cell death releases nuclear and cytosolic DAMPs such as HMGB1, which trigger inflammation [55]. In human cells, phosphorylated MLKL further engages SIGLEC12 to promote PMR, a species-specific mechanism absent in mice [56].

#### ZBP1: The Necroptotic Trigger

During IAV infection, necroptosis is predominately initiated by ZBP1, a nuclear innate immune sensor that recognizes Z-RNA structures generated during viral replication (Figure 1) [57]. ZBP1 interacts with RIPK3 through RHIM-RHIM domain interactions, forming a signaling platform that can initiate both necroptosis and apoptosis [58]. This ZBP1-driven mechanism is distinctive of IAV which replicates in the nucleus and is associated with nuclear envelope disruption and leakage of nuclear DNA into the cytosol.

Although ZBP1 is the dominant trigger, alternative pathways can activate necroptosis under specific conditions. Signaling through TNFR1 and Toll-like receptor (TLR) 3 and 4 can induce necroptosis when caspase-8 is inhibited or IAPs are depleted [59]. Viral proteins further modulate these responses. NS1 enhances MLKL oligomerization and membrane translocation and promotes NLRP3 inflammasome activation and IL-1β release [60]. In contrast, transforming growth factor-β-activated kinase 1 (TAK1) restrains premature necroptosis by suppressing RIPK1 activity through IκB kinase (IKK) signaling [61].

Consistent with this regulatory complexity, studies using MLKL-deficient mice or pharmacological inhibition of RIPK3 inhibitors during infection with mouse-adapted H1N1 PR8 have reported divergent outcomes [57,62,63]. In some models, *Mlkl*^-/-^ mice challenged with sublethal, and to a lesser extent lethal, doses of PR8 exhibit improved survival and reduced lung injury, supporting a pathogenic contribution of necroptotic signaling during influenza infection [57]. In contrast, other studies report that *Mlkl*^-/-^ animals display similar mortality to wild-type mice following sublethal infection, indicating that necroptosis is dispensable for disease outcomes under certain conditions [64]. Importantly, multiple studies demonstrate that RIPK3 can mediate host protection through MLKL-independent, caspase-8-dependent apoptotic signaling, explaining why disruption of RIPK3 and MLKL can yield non-overlapping phenotypes during influenza infection [58,63]. These discrepancies likely reflect differences in viral dose and strain, genetic background, redundancy with apoptotic pathways, and experimental endpoints assessed. Collectively, these findings underscore the context-dependent role of necroptosis during influenza infection and highlight the need for caution when extrapolating murine genetic studies to therapeutic targeting of necroptotic pathways.

### 2.4. Ferroptosis: An Emerging Contributor to IAV-Induced Lung Injury

Beyond caspase- and MLKL-dependent pathways, ferroptosis has emerged as a metabolically distinct form of PCD characterized by iron-dependent lipid peroxidation of polyunsaturated fatty acids (PUFAs), impaired glutathione peroxidase 4 (GPX4) activity, and excessive reactive oxygen species (ROS) generation (Figure 1). Ferroptosis is accompanied by unique mitochondrial ultrastructural changes, including mitochondrial shrinkage, increased membrane density, and outer mitochondrial membrane rupture, in the absence of nuclear condensation or fragmentation [65].

Recent studies implicate ferroptosis in virus-induced lung injury, including during IAV infection. In murine models, IAV infection increases lipid peroxidation markers such as malondialdehyde while reducing expression or activity of ferroptosis-related antioxidant defenses, most notably GPX4, in lung tissue [14]. Pharmacological inhibition of lipid peroxidation with ferrostatin-1 attenuates oxidative lung damage, reduces inflammatory cytokine production, and improves survival in models of severe influenza [14]. In addition, inhibition of indoleamine 2,3-dioxygenase 1 (IDO-1), a metabolic regulator linked to ferroptotic susceptibility, using 1-methyl-tryptophan ameliorates acute lung injury in IAV-infected mice. Collectively, these findings suggest that ferroptosis contributes to epithelial injury and disease severity during severe influenza.

### 2.5. Crosstalk and Functional Implications

Although individual PCD pathways are often studied in isolation, IAV infection reveals extensive functional overlap and regulatory interdependence among apoptotic and inflammatory cell death programs. Evidence from murine models indicates that apoptosis is generally protective [58,63], whereas pyroptosis, necroptosis, and ferroptosis are more frequently associated with immunopathology and severe disease outcomes [14,43,44,45,62,66,67]. However, these distinctions are blurred by extensive pathway crosstalk.

Caspase-3 exemplifies this interconnectivity by exerting opposing effects on pyroptotic execution (Figure 1). In specific contexts, caspase-3 cleaves GSDME to convert apoptosis into a lytic, inflammatory form of cell death, while simultaneously inactivating GSDMD through generation of an inhibitory p43 fragment [43,68]. Functional redundancy among gasdermins further complicates pathway assignment, as GSDME can compensate for loss of GSDMD [69], and conversely, GSDMD can partially substitute when GSDME is impaired [43].

Caspase-8 functions as a central regulatory node linking apoptotic and necroptotic pathways (Figure 1). Its inhibition diverts TNFR1 signaling toward RIPK3-dependent necroptosis, while its proteolytic activity blocks necroptosis by cleaving RIPK1 and RIPK3 even when pro-necroptotic signals are present. During IAV infection, ZBP1 activation can initiate both apoptosis and necroptosis concurrently, with caspase-8 serving as the decisive checkpoint. When caspase-8 is active, apoptosis predominates and when caspase-8 is inhibited, necroptosis ensues [57,58]. In murine macrophages, this interplay between caspase-8 and multiple death pathways contributes to the convergence of apoptotic, pyroptotic, and necroptotic features that has been described as an integrated cell death program termed PANoptosis [53,70]. However, evidence for PANoptosis during influenza infection derives predominately from mouse in vitro systems, and validation in human-relevant models remains limited.

Ferroptosis also intersects with other PCD pathways at multiple mechanistic levels [71]. ROS and mitochondrial dysfunction are shared features of ferroptosis and intrinsic apoptosis, suggesting overlapping upstream stress responses. In addition, lipid peroxidation products generated during ferroptosis can activate the NLRP3 inflammasome, providing a potential link between ferroptotic and pyroptotic signaling [72]. Whether ferroptosis operates sequentially, synergistically, or in parallel with other PCD programs during IAV infection remains unresolved.

Collectively, these findings define a network of partially redundant and temporally dynamic PCD pathways. The pathway crosstalk described above is not only mechanistic but also temporal, with cells transitioning between different death modalities over the course of infection. Accumulating evidence suggests that infected cells do not commit irreversibly to a single PCD pathway at the onset of IAV infection. Rather, the dominant pathway can shift as a function of viral burden, cellular stress thresholds, and the dynamic availability of pathway regulators.

Early in infection, when viral PAMPs are limited and cellular homeostasis is relatively intact, intrinsic apoptosis represents the predominant response, triggered by mitochondrial dysfunction and oxidative stress with minimal inflammatory consequences [23,73]. As infection progresses and viral replication intensifies, several factors drive transitions toward inflammatory PCD pathways. Cytosolic accumulation of viral Z-RNA species recognized by ZBP1 promotes necroptotic signaling through RIPK3 and MLKL [57]. Concurrently, inflammasome activation thresholds may be exceeded through K^+^ efflux, lysosomal damage, and mitochondrial ROS accumulation, shifting the balance toward pyroptosis [74,75].

The checkpoint function of caspase-8, described above, is central to this temporal transition. When caspase-8 activity is maintained, it blocks necroptosis even in the presence of ZBP1 activation by cleaving RIPK1 and RIPK3 [76]. However, as viral proteins accumulate and cellular stress intensifies, caspase-8 can be inhibited or degraded, thereby permitting necroptotic pathway engagement. Similarly, inflammasome priming and assembly requires time-dependent accumulation of danger signals, explaining the delayed onset of pyroptosis relative to apoptosis observed in many experimental systems.

This model of temporally dynamic pathway switching has important implications for therapeutic intervention. Early targeting of apoptosis may not prevent subsequent inflammatory PCD if viral replication continues unchecked. Conversely, interventions that prevent early apoptosis could paradoxically increase susceptibility to later necroptosis or pyroptosis. Understanding the kinetics and determinants of these pathway transitions will be essential for optimally timing host-directed therapies.

## 3. Cell Death Responses During IAV Infection in Human Cells

The PCD pathways described above have been identified predominately using cancer-derived human cell lines and murine models. Human respiratory tissues comprise diverse epithelial, myeloid, endothelial, and structural cell populations that differ markedly in susceptibility to IAV infection and in their preferential engagement of PCD pathways. However, cell-type-specific differences in viral tropism, baseline immune competence, and signaling thresholds strongly influence whether apoptosis, pyroptosis, or necroptosis predominates during infection. Moreover, in vitro human model systems vary widely in cellular origin, differentiation state, and expression of PCD machinery, all of which can profoundly shape experimental outcomes. Careful consideration of these model-specific features is therefore essential for accurate interpretation and translation to human disease.

Murine models remain indispensable for studying integrated immune responses, viral clearance kinetics, and survival outcomes that cannot be recapitulated in vitro. As discussed in the previous section, genetic knockout studies targeting RIPK3, MLKL, GSDMD, GSDME, and ZBP1 have been foundational for establishing causal roles for individual PCD pathways, experiments that are not currently feasible in human tissues. Accordingly, integrating murine in vivo systems with human ex vivo and in vitro models represents the most robust strategy for mechanistic insight and therapeutic development.

In the sections below, we examine PCD responses across major human respiratory cell types, highlighting dominant death pathways, strain-specific effects, and unresolved questions within each cellular context.

### 3.1. Cell Tropism and Receptor Distribution

Influenza infection is largely confined to the respiratory tract, where interactions among multiple epithelial, stromal, and immune cell populations collectively shape antiviral defense and inflammatory pathology. Defining which cell types are infected, and how each executes PCD, is therefore fundamental for selecting physiologically relevant experimental models.

#### 3.1.1. Receptor Distribution and Viral Entry

Viral entry is mediated by IAV hemagglutinin (HA) binding to sialic acid (SA) residues on the epithelial surface, and the receptor distribution of SA linkages strongly influences viral tropism and disease manifestation. Human-adapted influenza strains preferentially bind α2,6-linked SAs, which are enriched on ciliated and goblet cells in the upper respiratory tract, whereas avian strains favor α2,3-linked SAs that are more prevalent in the lower respiratory tract [77]. In primary bronchial epithelial cell cultures grown at the air-liquid interface (ALI), α2,6-linked SA is predominantly expressed on the apical surface, while basal cells primarily express α2,3-linked SAs [78]. This spatial organization has important implications for strain-specific infection patterns: human strains efficiently infect both ciliated and goblet cells, whereas avian strains exhibit more restricted tropism in the lung.

#### 3.1.2. Key Cell Populations

Epithelial cells lining the upper and lower airways are the principal site of viral replication. Alveolar macrophages (AMs) provide early sentinel defense within the airway lumen. Endothelial cells regulate vascular integrity, while fibroblasts coordinate tissue repair and remodeling. Both structural cell types contribute to inflammatory signaling and systemic complications during severe disease.

### 3.2. Epithelial Cells

Epithelial cells constitute the primary barrier to influenza and are the dominant site of viral replication. Beyond their barrier function, epithelial cells actively shape immune responses through cytokine and chemokine secretion [79,80]. Their PCD responses critically shape viral fitness, tissue injury, and immune activation. In vitro studies of epithelial cell death have relied primarily on three model categories: cancer-derived cell lines, primary cultures, and immortalized non-cancerous cell lines.

#### 3.2.1. Cancer-Derived Epithelial Cell Lines

Cancer-derived lung alveolar epithelial lines, particularly A549 and Calu-3, have been extensively used to study PCD mechanisms during IAV infection due their ease of culture and high transfection efficiency. However, their interpretive value is constrained by cancer-associated alterations, including impaired interferon signaling, altered metabolic thresholds, and dysregulated inflammasome pathways [81,82,83,84,85], which collectively distort innate and cell death responses. Consequently, these models may not accurately reflect either the magnitude or the inflammatory character of epithelial cell death during human infection (discussed further in Section 5.1).

Across numerous studies, infection of A549 and Calu-3 cells with diverse IAV strains, including pandemic and mouse-adapted H1N1 and highly pathogenic H5N1, consistently induces apoptosis. This is supported by reduced cell viability, caspase-3 activation, PARP cleavage, TUNEL positivity, and chromatin condensation [27,73,86,87,88]. Mechanistic analysis demonstrate that viral proteins such as NS1, PB1-F2, PA, and NP modulate apoptotic signaling through interactions with mitochondrial regulators and suppressing host anti-apoptotic pathways (Figure 1) [27,75,89,90]. Collectively, these findings establish apoptosis as a dominant outcome of IAV infection in cancer-derived epithelial cells.

In contrast, evidence for robust activation of lytic inflammatory cell death pathways in these models is limited. Markers of pyroptosis, including gasdermin cleavage, LDH release, and inflammasome-driven cytokine secretion, are generally weak or absent [49]. As a result, cell death in cancer-derived lines is typically dominated by apoptotic features, with minimal engagement of membrane-disrupting execution programs.

Emerging evidence also suggests that ferroptotic stress responses may occur in cancer-derived epithelial cells during IAV infection. In A549 cells, swine H1N1 infection increases ROS production, depletes glutathione, and promotes lipid peroxidation; inhibition of this process with ferrostatin-1 impairs virus replication and release [91]. However, because malignant cells exhibit altered iron handling, lipid metabolism, and redox homeostasis [92,93,94], these findings are difficult to interpret and require validation in physiologically normal human epithelial systems.

#### 3.2.2. Primary and Immortalized Non-Cancerous Epithelial Cells

Primary bronchial epithelial (PBEC) cultures and immortalized non-cancerous lines such as HBEC3-KT more closely recapitulate normal epithelial physiology and are increasingly recognized as superior models for studying influenza-induced PCD. These cells support robust replication of multiple IAV strains [43,51,80,95].

Recent studies using these systems reveal striking strain-specific differences in epithelial cell death responses. Seasonal and pandemic H1N1 and H3N2 strains differ in their ability to activate GSDMD, GSDME, and downstream pyroptotic events, such as LDH and cytokine release [43,44]. Across these strains, GSDME-mediated pyroptosis predominates as the lytic pathway, driven by caspase-3 activation, whereas inflammasome-dependent GSDMD activation is comparatively weak [43,51].

This dominance is reflected in both the timing and magnitude of effector activation. Cleaved GSDME and caspase-3 fragments appear earlier and reach higher levels than active caspase-1 or GSDMD fragments [43,51]. Notably, an inactive p43 GSDMD fragment (generated by caspase-3 cleavage) is also detectable early, further supporting caspase-3–mediated suppression of canonical GSDMD-driven pyroptosis. Epithelial cells express higher basal levels of GSDME than macrophages [45]. This enables caspase-3-driven GSDME activation to function as a rapid route to lytic cell death. Together, these findings support a model in which apoptosis initiates epithelial cell death but rapidly transitions into GSDME-dependent pyroptosis. Simultaneously, caspase-3 suppresses canonical inflammasome-driven GSDMD activation.

By contrast, necroptosis remains poorly defined in human epithelial cells. MLKL-dependent death has not been conclusively demonstrated in primary or immortalized non-cancerous human airway epithelium. This may reflect biological restriction of necroptosis in epithelial cells, higher activation thresholds relative to immune cells, or species-specific execution mechanisms (discussed in Section 5 and Section 6). Systematic interrogation of necroptotic signaling in primary epithelial systems is therefore needed.

Similarly, systematic evaluation of ferroptosis in human lung epithelial cells is lacking. Preliminary studies in BEAS-2B cells show time-dependent induction of ferroptotic marker (GPX4, SLC7A11), lipid peroxidation, iron accumulation, and glutathione depletion following mouse-adapted PR8 H1N1 infection [14]. Whether primary epithelial cells or HBEC3-KT undergo ferroptotic cell death, and whether ferroptosis contributes significantly relative to GSDME-mediated pyroptosis remains unresolved. Given that physiologically normal epithelial models revealed dominant GSDME signaling largely undetectable in cancer-derived lines, prioritizing these systems is essential for defining the relevance of ferroptosis to human influenza pathology.

### 3.3. Myeloid Cells

Beyond epithelial cells, myeloid populations, particularly AMs and infiltrating monocyte-derived macrophages, exhibit distinct PCD profiles that influence inflammatory outcomes. AMs are sentinel immune cells residing within the airway lumen and provide early defense against inhaled pathogens. Although human-adapted IAV strains can infect macrophages, they typically undergo abortive replication [79,96], rendering macrophages a functional dead end for viral spread. Nevertheless, AMs produce abundant cytokines [79,97] and eventually undergo cell death [98], contributing to the inflammatory response.

Notably, certain highly pathogenic avian influenza (HPAI) H5N1 strains can productively replicate in monocyte-derived macrophages and AMs [99,100], underscoring strain-specific differences in macrophage permissiveness and death responses. Although AMs comprise a relatively small fraction of total lung cells compared with epithelial populations, their loss may have disproportionately large consequences for immune regulation, viral clearance, and resolution of inflammation. In particular, inflammatory mediators and DAMPs released during lytic macrophage PCD may propagate epithelial injury through paracrine signaling, potentially exacerbating immunopathology.

#### Cancer-Derived Myeloid Lines and Human Blood-Derived Macrophages

Most in vitro studies of influenza-induced PCD in human myeloid cells rely heavily on peripheral blood mononuclear cells (PBMCs) [99,101,102,103] or the THP-1 monocytic leukemia cell line [53,60,104,105]. THP-1 cells are typically differentiated with phorbol 12-myristate 13-acetate (PMA) to generate macrophage-like cells for IAV infection. However, PMA concentration and exposure duration vary widely across studies, contributing to experimental heterogeneity [106].

Infection of macrophages with mouse-adapted PR8 H1N1 broadly induces mixed PCD, activating caspases-1/3/7/8; GSDMD, GSDME, and necroptotic mediators RIPK3 and MLKL [60,102]. IL-1β secretion and LDH release in infected macrophages suggest engagement of pyroptotic mechanisms, while partial inhibition of lysis by necrostatin-1 implicates necroptosis [101]. Avian H5N1 strains induce exaggerated cytokine production, variable inflammasome activation, and delayed apoptosis [37,104,107]. In contrast, studies of H3N2 strains largely emphasize apoptotic features, including ROS generation, and caspase-3 activation [99,105].

Despite this evidence, key questions remain unresolved. The relative contribution of individual PCD pathway to macrophage death, the temporal sequence of pathway activation, and the extent of compensatory signaling mechanisms when one pathway is inhibited are poorly defined. Moreover, whether primary human AMs isolated from bronchoalveolar lavage samples recapitulate PBMC- or THP-1-derived macrophage responses remains largely unexplored.

Direct comparisons across studies are further confounded by methodological variability in viral strain, multiplicity of infection, infection duration, and differentiation protocols influence cell death readouts. Standardized experimental frameworks and systematic side-by-side infections across multiple strains within the same cellular system are urgently needed to resolve these discrepancies.

### 3.4. Lung Structural Cells: Endothelial and Fibroblast Populations

Unlike epithelial cells, endothelial cells are typically abortively infected with IAV [108], with the notable exception of certain H5N1 isolates [109]. Nevertheless, endothelial cell, as well as fibroblasts, play critical roles in disease progression by regulating barrier integrity, cytokine signaling, and tissue repair.

#### 3.4.1. Endothelial Cells

Endothelial responses to IAV have been studied in a range of monoculture systems, including brain (HBMEC) [110], retinal (HRMEC) [111], umbilical vein (HUVEC) [111], and lung microvascular endothelial cells (HMVEC, HULEC) [109]. Most studies have reported compromised barrier integrity and/or increased inflammatory cytokine production following infection with H1N1 and avian H5N1 strains [110,112]. Endothelial dysfunction contributes to vascular leakage, pulmonary edema, and systemic inflammation, hallmarks of acute respiratory distress syndrome (ARDS) and fatal IAV infections (reviewed in [113]). During H5N1 infection, endothelial cells may contribute to hemorrhagic and systemic complications [109].

Evidence for influenza-induced PCD in endothelial cells remains limited. Annexin V positivity, TUNEL staining and caspase-3 cleavage indicate a capacity for apoptosis [114,115]. HMGB1 release [116] and transcriptomic signatures consistent with apoptotic or necroptotic activation [111,117] further suggest engagement of these pathways. These cells undergo PCD in response to viral components or inflammatory mediators, thereby exacerbating disease severity. However, direct demonstration of endothelial pyroptosis or necroptosis is lacking, identifying an important gap in current understanding.

#### 3.4.2. Fibroblasts

Studies of influenza-induced PCD in fibroblasts are sparse but indicate activation of apoptotic and pyroptotic signaling. One report describes activation of caspases-3/7/8 and GSDME cleavage in primary lung fibroblasts infected with mouse-adapted PR8 H1N1, consistent with combined apoptotic and pyroptotic activity [102]. Other studies demonstrate productive viral replication and inflammatory cytokine release in primary lung fibroblasts [80] and MRC-5 fetal lung fibroblasts [118] following H1N1 infection.

Although direct evidence for lytic PCD in structural cells remains limited, defining death pathways in endothelial and fibroblast populations is essential for understanding vascular leakage, interstitial remodeling, and strain-specific pathogenicity during severe influenza.

## 4. Advances In Vitro and Ex Vivo Models for Studying IAV-Induced Cell Death

While monoculture systems have been instrumental in defining cell-autonomous PCD mechanisms, they cannot capture the multicellular interactions that shape influenza pathogenesis in vivo. In the lung, infected epithelial cells release virions, cytokines, chemokines, and DAMPs that act on neighboring epithelial, endothelial, stromal, and immune cells. These interactions generate complex paracrine signaling networks and spatially restricted tissue injury that are absent in monoculture infections.

**Figure 2:**
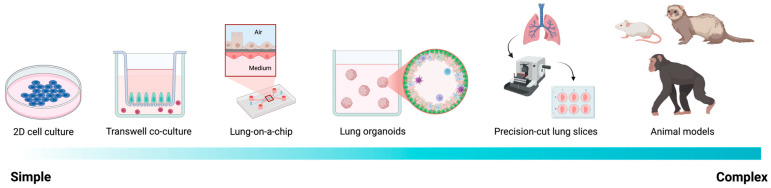
Experimental models for studying influenza virus infections across increasing levels of physiological complexity. Models range from 2D monocultures for reductionist mechanistic studies to Transwell co-cultures enabling epithelial-endothelial interactions at an air–liquid interface, lung-on-chip devices incorporating microfluidic flow and mechanical strain, 3D lung organoids recapitulating tissue architecture and cell diversity, precision-cut lung slices preserving native lung structure, and animal models capturing whole-organism physiology. The gradient reflects increasing physiological relevance, complexity, and experimental cost.

To address these limitations, increasingly complex and physiologically relevant human model systems have been developed, including epithelial-endothelial co-cultures, lung-on-chip platforms, 3D lung organoids, and precision-cut lung slices (PCLS). Figure 2 illustrates the continuum of experimental complexity from 2D monocultures to intact human tissue, while Table 1 summarizes the structural features, advantages, and limitations of each system. Collectively, these platforms preserve key elements of the human lung microenvironment and are well suited for examining strain-specific infection patterns, paracrine signaling, and the spatial dynamics of cell death.

However, as detailed in Table 1, relatively few influenza studies have leveraged these models to directly interrogate PCD pathways. Most investigations have prioritized viral replication kinetics and cytokine induction, rather than mechanistic dissection of PCD within intact tissue contexts. Expanding the application of these systems to examine PCD is therefore essential for understanding how cell death unfolds in situ and how epithelial, endothelial, and immune compartments collectively shape disease outcomes.

### 4.1. Co-Culture Studies

Dual-layer Transwell systems containing airway epithelial cells at the ALI and primary lung microvascular endothelial cells in the basolateral compartment emulate key features of the distal lung epithelium–vascular interface (Table 1). In these models, influenza infection of the epithelial layer consistently results in robust apical viral replication accompanied by basolateral transmission of inflammatory signals, even in the absence of direct endothelial infection [119]. Most studies report pronounced endothelial cytokine production (e.g., IL-6, CXCL10) and barrier dysfunction, measured via increased FITC-dextran permeability, despite abortive viral replication in endothelial cells [119,120]. These findings indicate that paracrine signaling, exposure to viral components, or epithelial-derived DAMPs, rather than direct infection, can drive endothelial activation and injury.

Comparable outcomes have been observed co-cultures using cancer-derived epithelial and endothelial cell lines infected with pandemic or mouse-adapted H1N1 strains and HPAI H5N1 viruses, including marked cytokine release and compromised epithelial barrier integrity [120,121]. However, systematic analysis of epithelial and bystander-cell PCD pathways, including identity of released DAMPs and their signaling consequences, remain limited in these systems.

Insights from SARS-CoV-2 co-culture models, including transcriptomic profiling has revealed epithelial apoptotic and mitochondrial stress signatures [122], suggest that similar multi-omic approaches could uncover previously unrecognized IAV-induced death pathways in co-culture contexts.

### 4.2. Lung-on-Chip Technology

Lung-on-chip platforms represent a substantial advance over static co-culture systems. These microfluidic devices recreate the epithelial ALI and vascular compartment using parallel channels separated by a porous, ECM-coated membrane (Figure 2 and Table 1) [123]. Importantly, they can also incorporate physiological biomechanical forces, including vascular shear stress and cyclic stretch that mimics breathing [124], that are absent in conventional cultures but can critically influence immune activation and tissue injury.

In influenza infection studies, lung-on-chip platforms co-culturing primary alveolar cells and pulmonary microvascular endothelial cells, when infected with H3N2 and H5N1, exhibit pronounced proinflammatory cytokine production and progressive loss of barrier integrity in both channels [125]. Bulk RNA-sequencing reveals divergent transcriptional responses, with epithelial cells upregulating antiviral, proliferative, and DNA replication programs, while endothelial cells preferentially induce ion-channel regulators and inflammatory pathways.

Notably, breathing-like cyclic stretch was shown to activate a receptor for advanced glycation end products (RAGE)-dependent innate immune pathway that restricts epithelial infection [125]. This finding highlights the importance of mechanical cues in shaping antiviral defenses and suggests that biomechanical forces may also modulate PCD pathway engagement, an underexplored area of influenza biology.

Integration of circulating immune cells further enhances model complexity. During late-stage infection with mouse-adapted H1N1 infection, PBMCs migrate toward the epithelial-vascular interface in parallel with peak cytokine production [126]. Such spatially organized immune-tissue interactions cannot be reproduced in static systems and underscore the utility of lung-on-chip platforms for studying immune-driven cell death.

More recently, fully immune-competent lung-on-chip models incorporating tissue-resident macrophages, dendritic cells, and circulating granulocytes within a functional microvascular network have been developed [127]. These systems recapitulate key features of severe influenza, including cytokine storm signatures that mirror those observed in hospitalized patient, and coordinated CXCL10 responses across epithelial and vascular compartments. Single-cell RNA sequencing identified stromal-immune CXCL12-CXCR4 interactions that limit tissue damage, illustrating that immune competency can modulate structural injury.

Lung-on-chip platforms have also enabled evaluation of antiviral drugs and CRISPR-based influenza therapies [128]. However, high costs, technological complexity, and limited throughput remain significant barriers to widespread adoption.

### 4.3. Lung Organoid Studies

3D lung organoids have rapidly become indispensable tools for modeling respiratory virus infection. Derived from induced pluripotent stem cells (iPSC) or primary human airway and alveolar tissues, organoids recapitulate key features of lung epithelium, including multicellular composition, ECM embedding, polarization, mucus secretion, and functional ciliation (Table 1) [129]. Relative to lung-on-chip platforms, organoid studies have focused primarily on cell-type vulnerability and cytokine responses rather than mechanistic analysis of PCD pathways.

Organoids derived from alveolar or bronchiolar lineages express both α2,6- and α2,3-linked SAs, permitting infection by human- and avian-adapted strains [130]. They also express ACE2 and TMPRSS2, enabling comparative studies across respiratory viruses such as SARS-CoV-2 [131]. Successful modeling of infection has been achieved with luciferase-expressing pseudotyped influenza particles inducing productive infection over 72 h [131], while mouse-adapted PR8 H1N1 infection caused delayed but sustained viral replication accompanied by robust cytokine induction [132]. Organoid infection with pandemic H1N1 (2009) achieves high viral titers (≥1000-fold increase) with strong type I and III interferon responses by 72 h post-infection [133].

A notable observation is the preferential loss of alveolar type I (AT1) cells following H1N1 infection [134]. Given that AT1 cells cover approximately 95% of the alveolar surface [135], their selective vulnerability could critically impair gas exchange, although the underlying death mechanisms remain poorly defined. A recent study implicated ferroptosis in primary human alveolar organoids following mouse-adapted H1N1 infection [14].

Emerging immune-competent organoid systems incorporating macrophages or iPSC-derived immune cells offer new opportunities to study multicellular interactions and immune-mediated epithelial injury [136,137]. Nevertheless, systematic mapping of apoptosis, pyroptosis, necroptosis, and ferroptosis within organoid models remains largely unexplored.

### 4.4. Precision-Cut Lung Slices

Human PCLS represent the most physiologically relevant ex vivo platform for studying respiratory virus infection. PCLS preserve native lung architecture, epithelial–endothelial interfaces, resident immune populations, and airway-alveolar organization (Figure 2 and Table 1). Typically, 150–500 µm thick, slices remain viable for 7–14 days following agarose inflation and vibratome sectioning [138,139,140].

PCLS support productive infection with avian H5N1 and seasonal H3N2, as well as other human respiratory viruses such as parainfluenza virus, rhinovirus, and SARS-CoV-2 [124,141,142,143]. Infection with IAV induces infectious virion release, robust cytokine production (e.g., IFNγ and IL-6), and LDH release indicative of lytic cell death. Immunofluorescence enables spatial characterization of infected foci and tissue-localized responses. Despite these advantages, detailed characterization of the specific PCD pathways engaged during influenza infection in PCLS remains scarce.

One donor-stratified study found age-dependent reductions in viral susceptibility, with older donors exhibiting reduced viral RNA loads [124]. This highlights the unique value of PCLS for interrogating how age, smoking history, environmental exposures, or comorbidities shape infection outcomes and cell-death responses, variables that are largely inaccessible in conventional cell culture.

## 5. Comparative Insights: PCD Profiling Across Human Model Systems

Having described human model systems from monocultures to complex tissue platforms, it is essential to directly compare how PCD responses differ across these platforms. Such comparisons reveal systematic differences in pathway engagement, kinetics, and magnitude that influence data interpretation and translational relevance. This section synthesizes how key experimental variables, including model origin, cellular composition, dimensionality, tissue complexity, and temporal scope, shape observed PCD outcomes during IAV infection. By highlighting recurring patterns and interpretive caveats, it provides guiding principles for experimental design and cross-model integration.

### 5.1. Model Origin: Cancer Versus Non-Cancer Monoculture

A landmark comparative study by Lee et al. (2018) directly assessed IAV-induced cell death across cancer-derived epithelial lines (A549, Calu-3) and non-cancerous epithelial models (BEAS-2B, NHBE) using mouse-adapted PR8 H1N1, revealing fundamental differences in both the magnitude and mode of cell death [49]. Under identical conditions, non-cancerous epithelial cells exhibited three- to five-fold greater cell death than A549 or Calu-3 cells. Moreover, the dominant death modality diverged markedly. Cancer-derived lines displayed predominantly apoptotic features, including caspase-3/7 activation and PARP cleavage, with minimal evidence of pyroptosis, whereas non-cancerous cells underwent sequential cell death, with early apoptosis transitioning to pyroptosis within 24–48 h, accompanied by IL-1β secretion and LDH release.

Subsequent mechanistic studies in normal epithelial systems indicate that this inflammatory phase is driven by caspase-3-driven GSDME activation (see Section 3.2.2) [43,51]. This execution pathway is weakly detected or absent in cancer-derived epithelial lines, consistent with malignant alterations in innate immune and PCD signaling pathways. As a result, cancer models fail to capture the apoptosis-to-pyroptosis transition that characterizes influenza-induced epithelial injury in human airways.

Comparable systematic comparisons between cancer-derived and primary endothelial or myeloid cells remain limited. However, one study suggests that THP-1 leukemia-derived macrophages are permissive to pro-inflammatory (M1) polarization but deficient in anti-inflammatory (M2) differentiation relative to PBMC-derived macrophages [144]. Consequently, cancer lines likely underestimate both the magnitude and inflammatory nature of cell death during human influenza infection.

### 5.2. Cellular Context: Monoculture Versus Multicellular Systems

In addition to model origin, epithelial monoculture systems isolate cell-intrinsic PCD mechanisms but lack the multicellular context required to model paracrine signaling and bystander injury. In these systems, epithelial apoptosis and pyroptosis are closely coupled to viral replication, typically initiating between 12 and 24 h post-infection as viral titers rise [43,51]. These models therefore excel at defining epithelial-autonomous death pathways but provide limited insight into multicellular disease processes.

By contrast, epithelial-endothelial co-culture systems reveal substantial bystander injury that is not apparent in monocultures. In Transwell models, apical infection of primary bronchial epithelial cells at ALI induces endothelial barrier dysfunction despite minimal or abortive endothelial infection [119]. Endothelial cells also exhibit increased permeability, elevated inflammatory cytokine production, and transcriptional stress signatures [119,120]. However, whether this dysfunction reflects endothelial apoptosis, pyroptosis, necroptosis, or non-lethal inflammatory activation remains unresolved.

However, systematic quantification of endothelial PCD pathways in co-culture studies remains limited. Most analyses focus on barrier integrity and cytokine output, with few studies directly assessing caspase activation, gasdermin cleavage, or MLKL phosphorylation in endothelial cells. This gap has important therapeutic implications, as interventions targeting epithelial PCD alone may fail to prevent vascular injury driven by paracrine signaling rather than direct infection.

### 5.3. Dimensionality: 2D Cultures Versus 3D Organoids

Beyond cellular context, 3D lung organoids reveal cell-type-specific vulnerability to IAV infection that is largely obscured in conventional 2D cultures. In 2D monolayers, uniform exposure to viruses, nutrients, and oxygen produces relatively homogeneous infection kinetics and cell death profiles, masking subtype-specific susceptibility. Parallel studies of SARS-CoV-2-infected lung organoids demonstrate enhanced infectivity and differential cellular responses in 3D systems compared with organoid-derived monolayers [145].

Kumar et al. (2011) demonstrated preferential depletion of alveolar type 1 (AT1) cells following H1N1 infection [134]. Given their critical role in gas exchange and limited regenerative capacity [135], selective AT1 loss likely has major functional consequences. The mechanisms underlying this vulnerability remain unclear and may involve differences in receptor distribution, baseline interferon signaling, antioxidant capacity, or cell-type-specific engagement of distinct PCD pathways. For example, whether AT1 cells are preferentially susceptible to ferroptosis, given their high metabolic demands and oxidative stress exposure, or differentially activate other death modalities remains underexplored.

Despite these advances, systematic mapping of PCD pathways within organoids remains limited. Most studies quantify viral replication and cytokine output without resolving which death pathways are activated in specific epithelial subtypes. Emerging single-cell, spatial transcriptomic, and proteomic approaches now make such analyses feasible but remain underutilized in influenza research.

### 5.4. Tissue Complexity: Engineered Systems Versus Native Lung Tissue

Complementing engineered systems, PCLS preserve native lung architecture and cellular diversity, revealing infection dynamics that differ fundamentally from simplified model systems. Whereas 2D cultures and organoids typically exhibit relatively uniform infection, PCLS develop spatially heterogeneous foci of infection interspersed with uninfected bystander regions [124,146]. These bystander regions may nonetheless exhibit inflammatory activation in the absence of productive infection.

These spatial gradients likely reflect distinct PCD programs. Cells within heavily infected foci may undergo rapid lytic cell death due to overwhelming viral burden, whereas peripheral cells may favor apoptosis in response to paracrine cytokines and DAMPs. More distant regions may experience inflammatory stress without overt cell death. However, these hypotheses remain untested and require direct experimental validation. Systematic spatial mapping of these responses using transcriptomic or proteomic approaches represents a largely unexplored area of influenza research with potential to redefine our understanding of tissue-level pathology.

### 5.5. Temporal Scope: Short-Term Versus Extended Culture Models

Independent of model complexity, the lifespan of experimental systems constrains the temporal phases of PCD that can be studied. Most cancer-derived and primary monocultures capture only early events (24–72 h post-infection), thereby missing later phases of sustained inflammation, tissue injury, and repair. In contrast, organoids and PCLS remain viable for 7–14 days, enabling interrogation of delayed, chronic, and resolution-phase responses [129,132,138,140,147]. Lung-on-chip platforms maintained at ALI can also be sustained for extended periods (2–3 weeks), and one influenza study demonstrating potential for epithelial and endothelial barrier regeneration over time [125].

These temporal differences have important implications for PCD pathway detection. Early-phase models primarily capture virus-driven cell death tightly coupled to active replication, whereas extended-duration systems may reveal secondary waves driven by paracrine inflammatory mediators, oxidative stress, and metabolic exhaustion. For example, pathways such as ferroptosis or delayed lytic death may only become prominent during later infection phases.

Extended-duration systems are therefore essential for capturing the full temporal evolution of epithelial injury and recovery. Emerging live-cell, spatial and longitudinal profiling technologies (see Section 7.2) further enhance the ability to resolve PCD dynamics across the course of infection.

## 6. Integrating Murine and Human Models: Divergences and Translation

Comparative analysis across human model systems reveals that the dominant pathways and execution mechanisms of influenza-induced PCD differ substantially from those inferred from murine studies. These divergences are not merely quantitative but reflect species-specific hierarchies in effector usage, pathway coupling, and terminal execution that directly shape immunopathology and therapeutic efficacy.

While murine models remain essential for causal genetic interrogation and whole-organism outcome measures, human epithelial systems increasingly demonstrate alternative PCD dominance patterns, particularly in pyroptotic and necroptotic execution, that challenge assumptions derived from mouse genetics. This section synthesizes these mechanistic mismatches, focusing on how differences in gasdermin usage, necroptotic execution, ferroptotic susceptibility, and viral strain adaptation constrain direct translation and necessitate coordinated cross-species validation. Table 2 provides a summary of key species-specific differences in PCD pathways, with detailed mechanistic discussion in the following subsections.

### 6.1. Pyroptosis: GSDMD vs. GSDME Dominance

A major species divergence lies in the relative contributions of gasdermin effectors to pyroptosis. In murine influenza models, both GSDMD and GSDME contribute to immunopathology in a strain-dependent manner. *Gsdmd*^-/-^ mice are protected against H1N1 and H3N2 infection [44,51], whereas *Gsdme*^-/-^ mice exhibit protection primarily against H3N2 and H7N9 [43,45,148]. Overall, GSDMD appears to be the more broadly engaged pyroptotic effector in mice, though GSDME plays important strain-specific roles.

In contrast, human airway epithelial cells display clear GSDME dominance. Primary human bronchial epithelial cells and HBEC3-KT lines exhibit robust caspase-3-dependent GSDME activation with comparatively weak GSDMD cleavage [43,51], resulting in characteristic apoptosis-to-pyroptosis transition (see Section 3.2.2). Available evidence suggests that this transition is less pronounced in the murine epithelium.

Additional species-specific differences in inflammasome activation are likely relevant. For example, NLRP1 is expressed and functional in human airway epithelial cells but is structurally divergent and largely non-functional in mice [149], highlighting an inflammasome pathway that is poorly captured in murine models. Although NLRP1 can engage caspase-1 in human epithelial cells, inflammasome activation in this context does not uniformly culminate in canonical lytic pyroptosis, and functionally significant GSDMD-mediated membrane rupture appears to be tightly constrained. Whether NLRP1 is engaged during influenza infection of human airway epithelial cells, and how any resulting inflammasome signaling would intersect with the prominent caspase-3-mediated inactivation of GSDMD observed in these cells, remains unresolved and warrants systematic investigation.

These species differences have important therapeutic implications. Strategies targeting NLRP3 or caspase-1 (described in Section 7) may demonstrate efficacy in murine models but be less impactful in humans, where GSDME predominates. Conversely, therapeutic approaches targeting GSDME or downstream apoptotic–lytic conversion pathways will require validation in human-relevant in vivo or ex vivo models to establish efficacy, pharmacokinetics, and safety.

### 6.2. Terminal Execution of Necroptosis

Necroptosis illustrates an additional species divergence at the level of PMR. In mice, phosphorylated MLKL oligomerizes to execute necroptosis [54], and genetic or pharmacological inhibition yields strain- and dose-dependent effects during influenza infection [57,62].

In humans, terminal necroptotic execution requires both MLKL and SIGLEC12, the latter of which is absent in mice [56]. This requirement suggests that human necroptosis is more tightly regulated and may not be effectively suppressed by MLKL-targeted therapies alone. Although phosphorylated MLKL has been detected in lung tissue from fatal human H7N9 infections [150], systematic characterization of necroptotic signaling in primary human epithelial cells, organoids, or PCLS remains limited.

### 6.3. Ferroptosis: An Emerging but Poorly Defined Pathway

Ferroptosis represents one of the greatest uncertainties in cross-species comparison. In murine models, mouse-adapted H1N1 infection increases lipid peroxidation, reduces GPX4 expression [14,151]. Pharmacologic inhibition of ferroptosis attenuates oxidative lung damage, reduces inflammatory cytokine production, and improves survival [14].

Human evidence remains sparse. A549 cells show oxidative stress, glutathione depletion, and lipid peroxidation during swine H1N1 infection, with partial rescue by ferrostatin-1 [91]. However, the interpretive value of these findings is limited by the cancer-derived nature of this model (see Section 3.2.1 and Section 5.1). Preliminary data from BEAS-2B cells and primary human alveolar organoids [14] show time-dependent lipid peroxidation, iron accumulation, and glutathione depletion following mouse-adapted PR8 H1N1 infection. However, systematic validation in primary bronchial epithelial cells, HBEC3-KT lines, or PCLS is lacking.

Single-cell RNA sequencing of nasal wash samples from IAV-infected individuals identified ferroptosis-associated gene signatures [14], and identified ferroptosis-related genes whose expression associated with severe disease [152]. Whether ferroptosis represents a dominant death pathway in humans, a secondary amplifier of injury, or strain-specific phenomenon remains unresolved.

### 6.4. PANoptosis and Pathway Redundancy

Crosstalk among PCD pathways can lead to PANoptosis, defined by the coordinated activation of apoptosis, pyroptosis, and necroptosis. In murine macrophages, PANoptosis is observed during experimental high-dose mouse-adapted influenza infection in the context of inflammasome priming [53]. In human macrophages, multiple PCD components, including caspases-1/3/7/8, GSDMD/E, RIPK3, MLKL, can be activated [102,105,107], though coordinated execution within individual cells has not been conclusively demonstrated.

Both species exhibit pathway redundancy, but compensatory routes differ. In human epithelial cells, loss of GSDME can promote compensatory GSDMD activation [43], while necroptosis inhibition can shift toward apoptosis [60,63]. Viral strain-specific flexibility further complicates therapeutic targeting, as inhibition of one pathway may redirect, rather than prevent, tissue injury.

### 6.5. Strain-Specific Responses: Divergent Patterns Across Species

Viral genetics and host receptor distribution strongly influence PCD responses, but experimental systems reveal species-specific patterns. Human airway epithelium predominantly expresses α2,6-linked SAs apically, whereas mice express both α2,6- and α2,3-linked receptors throughout the respiratory tract [77]. This difference shapes viral tropism: mouse-adapted strains, such as PR8 H1N1 acquired mutations that enhance replication in murine cells [79] and may consequently activate host signaling pathways absent during natural human infection, which limits direct translation.

In murine models, PR8 H1N1 causes severe disease and heightened inflammatory signaling, while seasonal strains induce mild pathology [79,153]. Genetic studies show strain-dependent reliance on individual PCD pathways, including differential engagement of pyroptotic effectors (see Section 6.1).

In human epithelial cells, seasonal and pandemic H1N1 and H3N2 isolates display pronounced strain-specific PCD profiles. Strains differ in their ability to activate GSDME and GSDME, induce LDH release, and drive inflammatory cytokine secretion [43,44]. The viral determinants underlying these differences remain incompletely defined. Specific mutations in NS1 or other gene segments may contribute, while variation in PB1-F2 is particularly complex given that some strains have truncated or absent PB1-F2 due to premature stop codons. These genetic differences likely contribute to strain-specific PCD pathway engagement [154,155], though the precise mechanisms remain to be fully elucidated.

Human tissue studies further reveal strain-dependent endothelial involvement. HPAI H5N1 can productively infect lung microvascular endothelial cells, which is not observed during infection with seasonal H1N1 and H3N2 viruses [109]. This tropism aligns with the vascular leakage and systemic complications observed clinically and is not captured by epithelial monoculture systems.

No single model captures the full spectrum of strain-specific PCD responses. Cancer lines obscure inflammatory cell death, primary epithelial cultures lack vascular context, and PCLS remain low throughput. Comprehensive profiling therefore requires parallel, multi-platform approaches.

### 6.6. Temporal Dynamics

Infection kinetics differ substantially between species and experimental systems, shaping both the timing and apparent dominance of PCD pathways. In murine models infected with lethal doses of mouse-adapted H1N1 and H3N2, peak viral titers and lung pathology typically occur at 3–5 days post-infection, coinciding with maximal inflammatory signal and immune-mediated tissue injury [79,156]. These kinetics compress early antiviral, inflammatory, and lethal phases into a narrow temporal window.

In contrast, human epithelial cell culture systems exhibit accelerated replication cycles, with productive infection peaking within 24–48 h [80], and apoptosis signaling often preceding overt inflammation. In human bronchial epithelial cells, apoptosis can transition into lytic, GSDME-mediated pyroptosis by 12–24 h, reflecting a rapid shift from non-inflammatory to inflammatory cell death that is less apparent in murine epithelium [43].

Extended human platforms, including organoids, lung-on-chip systems, and PCLS, may reveal additional temporal layers of pathology that are difficult to resolve in murine models. These include delayed epithelial and endothelial dysfunction, sustained cytokine production, secondary waves of bystander injury, and early reparative responses. Such systems also permit assessment of whether pathways such as ferroptosis or necroptosis emerge preferentially during prolonged inflammatory or oxidative stress phases rather than during peak viral replication.

These temporal and species-specific differences underscore the importance of using complementary model systems. Extended-duration human platforms can capture late-phase PCD events and pathway crosstalk that are compressed or absent in murine models, providing critical context for pathway-targeted therapeutic strategies. Integrating these insights with murine genetics and in vivo studies ensures both mechanistic understanding and translational relevance.

### 6.7. Translational and Therapeutic Implications

Effective translation of influenza-induced PCD biology requires integration of advanced human model systems with murine validation Human platforms (PCLS, organoids, co-cultures, lung-on-chip) resolve cell-type-specific and spatiotemporal features of PCD, whereas murine genetics enable causal dissection and evaluation of systemic outcomes.

The priority moving forward is to progress beyond descriptive infection and cytokine measurements toward quantitative, pathway-resolved mapping of apoptosis, pyroptosis, necroptosis, ferroptosis, and their crosstalk in physiologically relevant systems, followed by targeted in vivo validation.

#### 6.7.1. Clinical Insights from Human Models

Severe influenza-associated ARDS is characterized by extensive epithelial injury and barrier failure [157,158]. Human epithelial models demonstrate that apoptotic signaling can rapidly transition into GSDME-dependent pyroptosis (see Section 6.1), suggesting that the inflammatory phase of ARDS is driven by progression from apoptosis to lytic pyroptosis. Therapies that modulate downstream pyroptotic execution (e.g., GSDME-linked mechanisms) may therefore be more effective than upstream inflammasome blockade.

#### 6.7.2. Species-Specific Considerations

The species-specific differences summarized in Table 2 and detailed in Section 6.1, Section 6.2, Section 6.3, Section 6.4 and Section 6.5 necessitate a strategic approach to therapeutic development. Therapies targeting pathways that differ substantially between species, particularly GSDME-mediated pyroptosis and SIGLEC12-dependent necroptosis, require extensive validation in human primary cells, organoids, or PCLS using human-adapted viral strains before advancing to clinical development. In contrast, interventions targeting conserved pathways (e.g., core apoptotic machinery) may translate more directly from murine efficacy studies. Optimal therapeutic pipelines should integrate murine models for causal validation of pathway function with human-relevant platforms to confirm molecular targets, pathway regulation, and phenotypic outcomes match expectations. This integrated cross-species approach maximizes the strengths of each system while minimizing translation failures.

#### 6.7.3. Emerging Role of Ferroptosis

Murine studies support a pathogenic role for ferroptosis and demonstrate benefit from ferroptosis inhibition (see Section 2.4). In humans, evidence remains preliminary. Rigorous evaluation of ferroptotic biomarkers and functional readouts in primary human epithelial systems and PCLS is needed to determine whether ferroptosis acts as a primary driver or a secondary amplifier of injury. Given that GSDME-mediated pyroptosis was underappreciated in cancer lines and murine systems but dominant in human epithelium. Similarly, ferroptosis may exhibit species-specific prominence driven by differences in iron handling, lipid composition, and antioxidant capacity [159].

## 7. Therapeutic Development: Validation Framework and Therapeutic Development

Translating mechanistic insights into effective influenza therapies requires a structured validation pipeline. Candidate interventions are first evaluated in advanced human platforms, primary epithelial cultures, organoids, co-culture models, and PCLS, to map apoptosis, pyroptosis, necroptosis, and ferroptosis with temporal and cell-type resolution. Murine models then provide in vivo validation of causal pathways, systemic outcomes, and pharmacokinetics. Iterative integration of these systems ensures that mechanistic discoveries are both biologically meaningful and therapeutically actionable.

### 7.1. Limitations of Current Pharmacological Modulators

Current pharmacological modulators of PCD pathways outlined in Table 3, face significant limitations, including poor target specificity, off-target effects, pleiotropic immunomodulatory activity, and poor solubility that limits lung delivery, which collectively constrain clinical translation. With the exception of inflammasome inhibitors, which have been evaluated in influenza models [160,161,162,163], most of these compounds have not been systematically tested in the context of influenza infection in human-relevant models. Compounds targeting PCD pathways often lack validation in human-relevant lung models, to ensure efficacy and safety.

For example, RIPK3 inhibitors such as GSK’872 can induce cytotoxicity at higher concentrations due to on-target effect that paradoxically induce cytotoxicity via apoptosis, which has driven efforts to design safer, more selective compounds [164]. Similarly, gasdermin-targeting inhibitors, including disulfiram and dimethyl fumarate, act via nonselective cysteine modification and exhibit poor solubility that limits lung delivery, thereby constraining their clinical applicability [165,166,167]. Ferroptosis inhibitors, such as Ferrostatin-1, are hampered by limited bioavailability and poor solubility [168] and have been primarily validated in preclinical murine studies or cancer-derived systems rather than in primary human epithelium.

**Table 3:** Pharmacological Modulators of PCD Pathways.

Pathway	Inhibitor	Target	Status	Key Limitations
Necroptosis	Necrostatin-1 [169]	RIPK1	Preclinical	Off-target effects, limited specificity, poor solubility.
Necroptosis	UH15-38 [62]	RIPK3 kinase activity	Preclinical	May incompletely inhibit necroptosis, limited validation in human models.
Necroptosis	GSK-872 [59]	RIPK3	Preclinical	Cytotoxicity at higher concentrations; incomplete specificity.
Pyroptosis	Disulfiram [170]	GSDMD	FDA-approved (alcoholism)	Poor target selectivity, pleiotropic effects, poor solubility.
Pyroptosis	Dimethyl fumarate [165]	GSDMD/E	FDA-approved (multiple sclerosis)	Non-selective cysteine modification, pleiotropic immunomodulatory effects, poor solubility.
Pyroptosis	Necrosulfonamide [171]	GSDMD	Preclinical	Experimental tool compound, not clinically viable, poor solubility.
Inflammasome	MCC950 [172]	NLRP3	Preclinical	Liver toxicity, limited clinical translation.
Inflammasome	ADS032 [160]	NLRP1/3	Preclinical	Early-stage development.
Ferroptosis	Ferrostatin-1 [173]	Lipid peroxidation	Preclinical	Limited bioavailability, poor solubility.

### 7.2. Emerging Technologies for PCD Pathway Mapping

Recent technological advances address these challenges by enabling high-resolution mapping of PCD dynamics in human-relevant systems. Spatial transcriptomics preserves tissue architecture and identifies infected foci versus bystander regions with distinct PCD profiles [136,174]. Single-cell multi-omics, including RNA sequencing, proteomics, and epigenomics, captures heterogeneity in antiviral responses and identifies regulators of apoptosis, pyroptosis, necroptosis, and ferroptosis [175]. High-content live-cell imaging tracks caspase activation, membrane permeabilization, and mitochondrial stress over time in 2D cultures, organoids, co-cultures, and PCLS [176,177,178,179]. Functional genomics approaches, including CRISPR or RNAi in iPSC-derived epithelial and immune cells, provide causal evidence for regulators of influenza-induced cell death [180].

Combining these tools allows identification of which cells die, through which pathways, at what time points, and in response to which viral strains or paracrine signals. This informs biomarker development, patient stratification, and rational therapeutic design.

### 7.3. Integrated Validation Pipeline and Therapeutic Strategy

The pipeline begins with discovery in human-relevant systems, focusing on interventions that modulate pathway-specific PCD mechanisms, such as GSDME-dependent pyroptosis, SIGLEC12-dependent necroptosis, and ferroptotic stress. Promising candidates are then tested in murine models to assess in vivo efficacy, systemic pharmacokinetics, pharmacodynamics, and impact on survival and viral clearance. Iterative refinement between human and murine models optimizes dosing, timing, and combination strategies.

Clinical translation also requires consideration of pathway kinetics, though optimal therapeutic windows will vary by viral strain, inoculum-dose, and patient immune status. During early infection (approximately days 0–3), antiviral therapy may be prioritized while preserving beneficial apoptotic responses. In the peak inflammatory phase (approximately days 3–5), targeted inhibition of lytic PCD programs, such as GSDME-mediated pyroptosis [43,44], may be advantageous. During the resolution phase (days 7–14), interventions should support epithelial repair [158] without broadly suppressing PCD.

Proposed biomarkers, including GSDME N-terminal fragments, cell-free DNA, and emerging ferroptosis-associated signatures [152], can guide patient stratification and define optimal therapeutic windows. This integrated framework links mechanistic insights to actionable therapies, enabling selective limitation of inflammatory cell death while preserving protective antiviral functions.

## 8. Concluding Remarks

This review synthesizes evidence from human and murine systems to define how influenza virus infection engages PCD pathways, with important consequences for understanding disease pathogenesis and therapeutic translation. A central conclusion is that both the hierarchy and execution of PCD differ substantially across species and experimental models, necessitating careful consideration when extrapolating mechanistic findings.

In human airway epithelium, influenza infection preferentially engages caspase-3-driven, GSDME-mediated pyroptosis, in contrast to the canonical NLRP3-caspase-1-GSDMD axis that predominates in murine studies. Consequently, apoptotic signaling in human epithelial cells rapidly transitions into inflammatory lytic death, a process underrepresented in cancer-derived cell lines and is less well-documented in murine epithelium.

Human necroptosis also exhibits distinct regulatory features, including dependence on SIGLEC12 for terminal execution, further limiting direct translation from murine models. Ferroptosis has emerged as a potential contributor to influenza-associated lung injury; however, its quantitative importance relative to inflammatory pyroptosis in human airway epithelium remains unresolved.

Model selection strongly shapes observed PCD outcomes. Cancer-derived epithelial lines attenuate inflammatory cell death and obscure key transitions characteristic of physiologically normal epithelium. Primary human airway epithelial cells and non-cancerous immortalized lines reveal strain-specific PCD hierarchies and temporal dynamics not captured in transformed models. Increasing system complexity provides additional resolution: co-culture platforms uncover epithelial–endothelial paracrine injury, organoids enable cell-type-specific vulnerability analyses, and patient-derived lung slices preserve native architecture and donor-specific responses essential for translational validation.

Despite these advances, critical gaps remain. Necroptosis in human epithelial cells is incompletely defined, ferroptosis requires systematic validation in primary human airway models, and spatially resolved mapping of PCD within intact tissue contexts remains limited. Strain-comparative studies are scarce, and influenza B virus remains notably understudied across PCD pathways. Addressing these gaps is essential for predicting pathogenicity and guiding host-directed therapeutic development.

Effective translation therefore depends on bidirectional validation. Murine models remain indispensable for establishing causality and assessing systemic outcomes, but species-specific execution mechanisms require confirmation in human-relevant platforms prior to clinical application. Current pharmacological modulators of PCD pathways exhibit limited specificity and substantial off-target effects, underscoring the need for next-generation inhibitors evaluated in physiologically relevant human systems. Successful host-directed therapies must preserve protective apoptosis while selectively limiting inflammatory cell death, align with disease-phase-specific kinetics, and account for the human-specific apoptosis-to-pyroptosis transition mediated by GSDME.

Emerging technologies, including spatial transcriptomics, multiplexed imaging, live-cell reporters, and functional genomics, now enable precise mapping of when, where, and how distinct PCD pathways are engaged during influenza infection. Explicitly accounting for species differences and model-specific constraints will be critical for translating mechanistic insight into therapies that reduce immunopathology while preserving antiviral defense, thereby improving preparedness for both seasonal and pandemic influenza.

## Figures and Tables

**Table 1:** Comparative analysis of experimental models for studying influenza-induced PCD.

Model	Cell Types	Structural Aspects	Advantages	Disadvantages	PCD Findings
Cancer-derived cell lines	A549, Calu-3.	2D monolayer.	Ease of culture and genetic manipulation. Low cost. Scalable for high-throughput screening.	Poor representation of normal airway physiology. Altered cell death and innate immune signaling. Limited ability to model multicellular/tissue-levels responses. Usually lacking mucus secretion and ciliary function.	Apoptosis via caspase-3/8/9, PARP cleavage. Human cancer lines are more severely impaired in the NLRP3/GSDMD signaling axis. Ferroptosis markers (ROS, lipid peroxidation, glutathione depletion) in A549 cells, but altered iron/lipid metabolism in cancer cells limits relevance.
Primary/immortalized non-cancer	PBECs, HBEC3-KT, BEAS2B.	2D or ALI.	Physiologically normal. Donor-specific studies.	More difficult and costly to culture.	Strain-specific apoptosis and pyroptosis. GSDME-dominant over GSDMD. p43-GSDMD inactivation via caspase-3. Temporal transition from apoptosis to pyroptosis. Ferroptosis reported in BEAS-2B.
Co-culture (Transwell)	2D co-culture of epithelial, stromal, or immune cells separated by a synthetic membrane.	ALI. Mucin production.	Easy setup. Partial intercellular interactions. Measures barrier integrity. Moderate scalability.	Limited cellular heterogeneity. Cannot model dynamic immune recruitment. Lacks full tissue complexity and dynamic mechanical forces.	Epithelial PCD triggers paracrine endothelial activation and barrier dysfunction without direct endothelial infection. PCD mechanisms in bystander cells remain undefined.
Lung-on-Chip	2D co-culture of epithelial, stromal, and immune cells in a microfluidic chip.	ALI Mimics vascular flow and breathing (cyclic stretch). Mucin production.	Integrates fluidic and mechanical features. Partial intercellular interactions. Real-time analysis. Simulates drug kinetics. Supports long-term infection.	Requires specialized equipment and expertise. Limited throughput. High cost.	Mechanical stretch activates RAGE-dependent restriction of infection. PCD pathways are not well characterized.
Lung organoid	3D culture of differentiated epithelial and stromal cells.	Polarized epithelium. Cilia pulsation. Mucin secretion. Matrigel-based ECM.	High cell heterogeneity. Mimics lung architecture. Derived from patient-specific iPSCs or tissues. Suitable for long-term infection.	Absence of immune cells. Inward-facing apical surface complicates infection. Batch variability. High cost. Suitable for long-term infection.	Preferential AT1 cell loss post-H1N1 infection. Robust cytokine responses. Ferroptosis reported in human alveolar organoids. Specific PCD mechanisms not defined.
Ex vivo Lung slices	Native tissue section containing epithelial, structural, immune cells.	Native ECM and cellular spatial architecture. Airway branching and vascular networks.	Preserves tissue complexity. Spatial dynamics of infection. Donor-specific studies. Physiologically relevant drug testing. Adaptable across species.	Needs fresh tissue and specialized preparation. Low throughput. Short-term viability (7–14 days). Donor and slice variability. Ethical constraints.	LDH release indicates lytic death. Focal infection patterns. Age-dependent viral susceptibility. PCD pathway analysis lacking.
Murine Models	Whole organism.	Native tissue.	Genetic manipulation. Survival endpoints. Disease correlations. Establishes causality that is impossible in humans.	Species differences in PCD machinery and IAV receptors. Inbred strains lack genetic diversity strains seen in humans.	Mouse-adapted IAV strains may not mimic human IAV. Apoptosis is generally protective/anti-viral. GSDMD/E knockout improves survival. Necroptosis dispensable or context dependent. Ferroptosis inhibition improves survival.

**Table 2:** Key species-specific differences in IAV-induced PCD.

PCD Pathway	Mouse Models	Human Models	Implication
Apoptosis	Caspase-3, mitochondrial pathway conserved.	Caspase-3, mitochondrial pathway conserved.	Likely translates well.
Pyroptosis	Strain-dependent: GSDMD (H1N1, H3N2), GSDME (H3N2, H7N9).	GSDME dominant in epithelium (caspase-3-driven).	GSDMD-targeted therapies may be less effective in human epithelium.
Necroptosis	RIPK3 → MLKL → membrane rupture.	RIPK3 → MLKL + SIGLEC12 → membrane rupture.	MLKL inhibitors alone may be insufficient in humans.
Ferroptosis	Well-documented.	Preliminary evidence in A549, BEAS-2B.	Requires validation in human primary cells.
PANoptosis	Demonstrated in macrophages (high MOI).	Multiple pathways activated; coordinated execution unclear.	Mechanism needs clarification in human cells.

## Data Availability

No new data were created or analyzed in this study. Data sharing is not applicable to this article.

## Abbreviations

The following abbreviations are used in this manuscript:

ALI	Air–liquid interface
AM	Alveolar macrophage
APAF1	Apoptotic protease-activating factor 1
ARDS	Acute respiratory distress syndrome
AT1/2	Alveolar type 1/type 2 cells
CRISPR	Clustered regularly interspaced short palindromic repeats
DAMP	Damage-associated molecular pattern
DISC	Death-inducing signaling complexes
ECM	Extracellular matrix
FADD	Fas-associated death domain protein
FasL	Fas ligand
GPX4	Glutathione peroxidase 4
GSDMD	Gasdermin D
GSDME	Gasdermin E
HA	Hemagglutinin
HPAI	Highly pathogenic avian influenza
HSP70/90	Heat shock proteins 70/90
IAP	Inhibitor of apoptosis
IAV	Influenza A virus
IDO1	Indoleamine 2,3-dioxygenase 1
iPSC	Induced pluripotent stem cells
LDH	Lactate dehydrogenase
LUBAC	Linear ubiquitin chain assembly complex
M1	Matrix protein 1
MLKL	Mixed lineage kinase domain-like protein
NA	Neuraminidase
NINJ1	Ninjurin-1
NLRP3	NOD-, LRR-, and pyrin domain-containing protein 3
NP	Nuclear protein
NS1	Nonstructural protein 1
PAMP	Pathogen-associated molecular pattern
PARP	poly(ADP-ribose) polymerase
PB1-F2	Polymerase basic protein 1 frame 2
PBEC	Primary bronchial epithelial cell
PBMC	Peripheral blood mononuclear cells
PCD	Programmed cell death
PCLS	Precision-cut lung slice
PMR	Plasma membrane rupture
PR8	Mouse-adapted A/Puerto Rico/8/1934 (H1N1) strain
RAGE	Receptor for advanced glycation end products
RHIM	RIP homotypic interaction motif
RIPK	Receptor-interacting protein kinase
ROS	Reactive oxygen species
SA	Sialic acid
SIGLEC12	Sialic acid-binding immunoglobulin-like lectin 12
ssRNA	Single-stranded ribonucleic acid
TRADD	TNFR1-associated death domain protein
TRAIL	TNF-related apoptosis-inducing ligand
ZBP1	Z-DNA binding protein 1
